# Concentrating Cocoa Polyphenols—Clarification of an Aqueous Cocoa Extract by Protein Precipitation and Filtration

**DOI:** 10.3390/membranes14110242

**Published:** 2024-11-17

**Authors:** Nicole Beeler, Tilo Hühn, Sascha Rohn, Renato Colombi

**Affiliations:** 1 Research Group Food Process Development, School of Life Sciences and Facility Management, Institute of Food and Beverage Innovation, ZHAW—Zurich University of Applied Sciences, 8820 Wädenswil, Switzerland; tilo.huehn@zhaw.ch; 2 Department of Food Chemistry and Analysis, Institute of Food Technology and Food Chemistry, Technische Universität Berlin, 13355 Berlin, Germany; rohn@tu-berlin.de; 3 Oro de Cacao AG, Chocolate Manufacturer, 8807 Freienbach, Switzerland; renato.colombi@orodecacao.com

**Keywords:** cocoa extract, cocoa flavanols, precipitation, filtration, clarification

## Abstract

The seeds of *Theobroma cacao* L. are rich in antioxidant flavonoids such as flavan-3-ols, which are valued for their health benefits. In this context, it is of interest to improve flavanol content in cocoa extracts. The present study aimed at improving the clarification process of an aqueous cocoa extract using protein precipitation and filtration. Five pH modifications and two bentonite amounts were tested for their effects on protein precipitation and flavanol content. Micro- and ultrafiltration as a subsequent step was done by testing three different ceramic membranes (30, 80, and 200 nm). Lower pH in pre-treatment reduced protein content and kept flavanols constant, while at higher pH, flavanols were reduced up to 40%. Larger membrane pores enhanced polyphenol permeation, while smaller pores limited protein permeation. Adjusting pH to the isoelectric point increased protein adsorption, improving filtration quality despite decreased permeate flux. However, membrane fouling results in higher permeate quality due to increased selectivity. Furthermore, the addition of bentonite during filtration reduced both protein and flavanol content in the permeate, similar to the effects seen in the pre-treatment of the supernatant. Optimizing pH and membrane pore size enhances the recovery and quality of polyphenols during filtration, balancing protein removal and flavanol retention.

## 1. Introduction

Bioactive compounds in various plant foods have attracted considerable interest in recent decades due to their health-promoting properties [1,2,3]. The seeds of the plant *Theobroma cacao* L. are quite prominent, as they are very rich in antioxidant polyphenols, with the majority being flavonoids, especially flavan-3-ols, and their oligomers. The latter are also called procyanidins. They have different degrees of polymerization (DP). In cocoa, about one-third of the flavan-3-ols are the monomeric (+)-catechin and (−)-epicatechin, while the procyanidins make up the rest [4]. Accordingly, chocolate and cocoa-containing products are consequently a source of bioactive polyphenols with potential health benefits as well [3,4]. In addition to health-promoting effects such as antioxidant and anti-inflammatory properties, cocoa is also reported to have prebiotic effects, in which it promotes the growth of beneficial bacteria in the colon [3]. However, for trying to make processes more sustainable and (food) products healthier, different approaches for adding value have been investigated for several years, focusing on the recovery and/or purification of bioactive substances. Polyphenols, in particular, have gained importance due to their various applications in the food industry as well as in cosmetics and pharmaceuticals [1,2,3]. However, with regard to cocoa, cacao beans also contain approximately 10–15% protein based on the dry weight [5,6,7]. Although both compound classes—polyphenols and proteins—are highly appreciated, they bear a severe challenge for either the protein or the phenolic compound purification and enrichment processes, as different interaction mechanisms make it difficult to separate proteins and phenolic compounds from each other [8]. In reversible interactions, non-covalent bonds such as hydrogen bonds, hydrophobic bonds, van der Waals forces, and electrostatic interactions are the basis of quite stable complexes [9,10,11]. These interactions are further influenced by various factors, e.g., the structure of the phenols and proteins, the pH value, the temperature, and the ratio of proteins and polyphenols in the environment [8].

For several years, membrane technology has been a widely studied approach to the purification and enrichment of polyphenols. It gained importance not only in the food industry for adding value to waste and by-products but also in the beverage industry as an alternative to traditional thermal processes for recovering, fractionating, and concentrating various ingredients from aqueous and alcoholic products and by-products [1,12,13]. This is because the processing methods used in the beverage industry, such as evaporation and rectification, often lead to thermal and oxidative degradation of sensitive ingredients such as polyphenols, resulting in a diminished quality [14], which then also leads to reactions, forming covalently bound adducts between the polyphenols and especially proteins. Several studies showed that the use of high temperatures (>65 °C) leads to a significant oxidative decomposition of polyphenols [15] with subsequent degradation products but reaction product formation [16].

Cissé et al. [17] showed that the enrichment of polyphenols by membrane technology is desirable, as such a process leads to a lower thermal impact on thermolabile compounds. Other advantages of membrane technology include processing without intense pH influence, separation of substances in their native form, and significant energy savings [18,19]. This is important because phenolic compounds polymerize to melanins or crosslink proteins in an alkaline solution and form reaction products with different solubility, (brown) color, or further properties [20].

In order to achieve maximum purification, microfiltration is often followed by a diafiltration step using water as a solvent. For example, some studies showed that the use of crossflow microfiltration (in some studies, even with diafiltration) could clarify and stabilize fruit juices or separate as well as concentrate bioactive compounds [21,22,23]. However, membrane fouling is one of the main problems in filtration due to the accumulation and deposition of particles on the membrane surface, which affects the productivity of the process. Fouling can be controlled by pre-treatment of the educt. In addition to centrifugation, precipitation by pH-modification and fining agents (e.g., bentonites) are important pre-treatment methods, where the removal of suspended solids has a major influence on membrane fouling and filtration kinetics [24].

There is limited information on the use of pre-treatment with pH-modification and bentonite addition in combination with micro- and ultrafiltration to clarify the aqueous cocoa extract and enrich the flavanol content of cocoa extracts. As many of the polyphenols change their polarity when being protonated under acidic conditions, pH conditions can significantly affect adsorption or interaction behavior towards the other compounds, such as proteins [7], but also membrane surfaces, depending on the membrane material used. The fining agent is able to bind and occlude proteins and has its optimum environmental pH value at pH 3.0–3.3 [25,26]. A disadvantage of bentonite treatment is a potential decrease in polyphenols associated with protein removal due to the adsorption of the polyphenols to the proteins [27], but it might be necessary at all to get rid of proteins and further compounds for enabling a higher concentration of polyphenols at all. However, binding and the interactions behind it are quite complex and can occur in the different steps of the whole process, from extraction over precipitation to filtration.

Nonetheless, it is hypothesized that the mix of techniques significantly influences the yield of flavanols but can be optimized to a certain extent. Consequently, the aim of the present study was to investigate different process approaches for the purification and enrichment of flavanols in cocoa extract. In this study, two process steps were investigated: *(I)* protein precipitation by pH adjustment and bentonite addition followed by centrifugation and *(II)* filtration of the supernatant using a dynamic crossflow filtration laboratory system. The extracts were characterized in terms of dry matter, protein, and cocoa flavanol content, with the latter expressed as the sum of oligomeric procyanidins of different degrees of polymerization (DP1-7). Filtration productivity was evaluated with regard to permeate flux.

## 2. Materials and Methods

### 2.1. Chemicals and Reagents

Bentonite (high-quality Ca-Na-bentonite granules) for the pre-treatment experiments was purchased from Erbslöh Geisenheim GmbH (Geisenheim, Germany). Sodium hydroxide (1 N), anhydrous citric acid (≥99.5%), and absolute ethanol (0.79 kg/L) were purchased from Sigma-Aldrich Chemie GmbH (Buchs, Switzerland). For Bradford protein analysis, the Coomassie Plus (Bradford) Protein Assay Kit and standard protein bovine serum albumin (BSA) were acquired from Thermo Fisher Scientific Inc. (VWR International GmbH, Dietikon, Switzerland).

In addition, the following chemicals were used for flavanol extraction and analysis (all HPLC grade): Acetone, acetonitrile, acetic acid (glacial), and methanol (Sigma-Aldrich Chemie GmbH, Buchs, Switzerland). Pure water used for the experiments and analysis was produced with the water purification system of Merck Milli-Q (0.22 µm) (Simplicity UV, Merck & Cie, Schaffhausen, Switzerland). Cocoa Extract Calibrant (NIST Reference Material No. 8403) was purchased from the National Institute of Standards and Technology (NIST, Gaithersburg, MD, USA). It was used to prepare standards for identification and quantification.

### 2.2. Physicochemical Analysis of Extract, Supernatant, and Permeate

To verify the filtration performance, the following analyses were carried out: solids content as dry matter, protein content as BSA, and flavanol content as degree of polymerization (DP1-7).

#### 2.2.1. Dry Matter Content Based on a Halogen Dryer Method

The dry mass was measured by means of a halogen dryer (Moisture Analyzer HC103, Mettler Toledo GmbH, Greifensee, Switzerland), according to the method described by Beeler et al. [28]. For the alignment of the method for the halogen dryer, the weighing pans were pre-dried for 1 h in a drying oven (FD-S 115, Binder GmbH, Tuttlingen, Germany) at 103 ± 2 °C and then cooled for 1 h in a desiccator. After noting the exact weight of the dish, the sample was weighed, recorded, and dried for 4 h in the same oven. After cooling in the desiccator, the weighing pan with the sample was reweighed. To ensure that equilibrium was achieved, the drying procedure was repeated for 1 h and checked to see if the reweighed weight remained unchanged.

#### 2.2.2. Protein Content Using the Bradford Method

The Bradford method was performed using the Coomassie Plus (Bradford) Assay Kit, according to Bradford [29]. For this, 0.05 mL of the sample was mixed with 1.5 mL of Coomassie reagent in a vial and incubated for 10 min at room temperature. The sample solution was then measured against water as a blank at a wavelength of 595 nm using a UV/Vis spectrophotometer (Genesys™ 10S, Thermo Fisher Scientific AG, Reinach, Switzerland). To determine the protein concentration of each sample, a calibration curve was prepared according to the standard test tube protocol (working range 100–1500 µg/mL) and measured at 595 nm. A third-order polynomial regression line of $y=0.2698\times x^{3}-0.1076\times x^{2}+0.8758\times x+0.0025$ and $R^{2}=0.9997$ was obtained. BSA was used as a calibration standard, and results were expressed as milligrams of BSA equivalent per gram of dry matter (mg BSA/g dm).

#### 2.2.3. Content of Individual Flavanols Using Ultra-Performance Liquid Chromatography (UPLC) Analysis

The flavanol content was determined using the official AOAC 2020.05-20 described by Bussy et al. [30,31] for the determination of flavanol and procyanidin oligomers (DP1-7) in cocoa-based products. The flavanols and procyanidins were extracted with an acidified aqueous acetone solvent system (AWAA; acetone:water: acetic acid, 70:30:1, *v*/*v*/*v*). The extracts were then filtered through a 0.22 µm syringe filter (VWR^®^ Syringe Filter, hydrophobic PTFE, 13 mm, 0.22 µm, VWR International GmbH, Dietikon, Switzerland) and transferred to chromatography vials for HPLC analysis.

*Extraction*—For the extraction of flavanols and procyanidins from the cocoa extract, 0.5 mL of the sample was pipetted into a 5 mL disposable centrifuge tube, and 4.5 mL of AWAA was added. The solution was shaken briefly by hand, and then the tube was placed in a sonic bath at 50 °C for 5 min. The sample was then centrifuged at 3068× *g* for 5 min. The extracted samples of cocoa extract from Peru were further diluted with AWAA at a ratio of 1:3, and those from Cuba and the Dominican Republic at a ratio of 1:2. Permeate samples from Peru were diluted directly with AWAA at 1:30, and those from Cuba and the Dominican Republic at 1:20. Approximately 1 mL from the diluted cocoa extract and permeate was then transferred through a 0.2 µm syringe filter into an HPLC vial.

*Chemical analysis*—Compound composition was determined using ultra-performance liquid chromatography with fluorescence detection (UPLC-FLR) on a Waters acquity UPLC system with a sample manager (FTN-H) and a quaternary solvent manager (QSM) system coupled to a Waters acquity UPLC-FLR detector (Waters AG, Baden-Dättwil, Switzerland). The injection volume was 2 µL.

*Chromatographic conditions*—The chromatographic column was a torus diol (100 × 3.0 mm id, 1.7 µm, 130 Å particle size, Waters AG, Baden-Dättwil, Switzerland). The flow rate was set to 1 mL/min, and the column temperature was maintained at 50 °C. Set and maintain the autosampler at 5 °C. It may be necessary to equilibrate the column with 50/50 solvent A/solvent B for at least 10 min before analysis.

*Solvents and gradient*—The two mobile eluents consisted of acetonitrile-acetic acid (98 + 2, *v*/*v*) (mobile phase A) and methanol-water-acetic acid (95:3:2, *v*/*v*/*v*) (mobile phase B).

HPLC gradient: 0.37 min, 0% B; 10.03 min, 45% B; 0.25 min, 95% B; 2.35 min, 95% B; 0.1 min, 0% B. The total run time was 13.10 min, and the total run equilibration was 3 min.

*Fluorescence detection*—Detection was performed by fluorescence detection using an excitation wavelength of 230 nm and an emission wavelength of 321 nm.

*Quantification*—The cocoa extract calibration standard (NIST Reference Material No. 8403) was used to determine retention times and to establish calibration lines. A linear regression was used to calculate the content of each DP group. The software Empower 3 (Waters AG, Baden-Dättwil, Switzerland) was used to integrate and calculate the flavanol content using the regression line. The results are expressed as mg/g dry matter (dm).

### 2.3. Experimental Design of the Clarification of the Cocoa Extract

**Figure 1:**
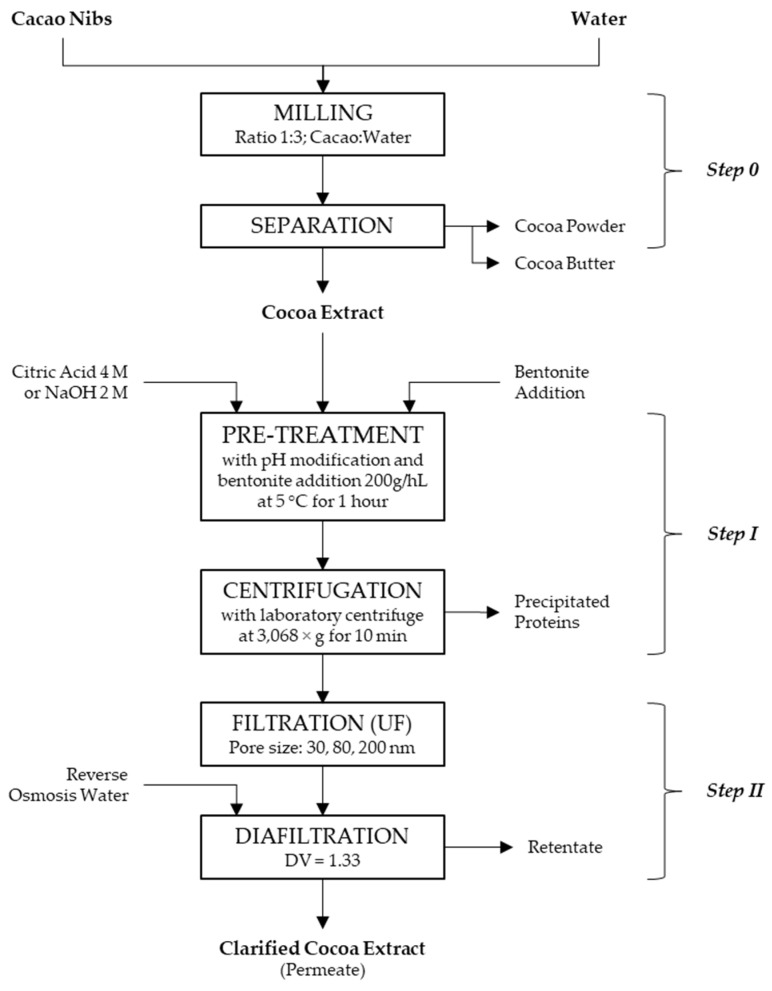
Schematic diagram of the clarifying process of the cocoa extract with the extraction, pre-treatment, and filtration step.

Clarification of an aqueous cocoa extract was carried out in different steps: *step (0)* extraction of aqueous cocoa extract using the patented cold extraction process, developed by Oro de Cacao AG, Freienbach, Switzerland, *step (I)* precipitation of the proteins by pH adjustment and bentonite addition with subsequent centrifugation, and *step (II)* filtration of the supernatant with a dynamic crossflow filtration laboratory system (crossflow filtration with rotating disc module; CRDM) from Novoflow GmbH (CRD-01-152-0.016, Novoflow GmbH, Rain, Germany). However, the extraction *step (0)* was not further optimized in the present study as the aqueous cocoa extract used in the present experiments was provided by Oro de Cacao AG. A simplified scheme of the proposed procedure to concentrate the flavanols in the cocoa extract obtained from cacao nibs can be found in Figure 1.

#### 2.3.1. Extraction of Cocoa Extract—*Step (0)*

As mentioned above, the aqueous cocoa extract was provided by Oro de Cacao AG. The aqueous cocoa extract was prepared in the same way as described in a previous study by Beeler et al. [28]. Briefly, it was obtained by grinding debacterized, unroasted cacao nibs with water at a ratio of 1:3. The resulting mixture, known as slurry, was then heated to 65 °C and separated into a solid, an oil, and a water phase using a 3-phase decanter (industrial scale: capacity 4 m^3^/h). For the experiments, the origin of the cacao nibs in Cuba, Peru, and the Dominican Republic were used. A regular production, as used for these experiments, was produced with a continuous flow rate of 500 kg/h of cacao nibs. The water phase obtained was then further purified using a centrifuge (industrial scale: capacity 2.5 m^3^/h) in order to further reduce the content of solid matter and fat in the phase [32]. The purified water phase, known as cocoa extract, contains proteins, carbohydrates, and fiber, as well as water-soluble compounds such as polyphenols and alkaloids [33]. The amount of cocoa extract used in the experiments was taken directly from the daily production of the cocoa processing plant and stored at −20 °C in 20 L-buckets.

#### 2.3.2. Precipitation and Centrifugation of the Cocoa Extracts—*Step (I)*

The pH of the cocoa extracts was modified, and bentonite was added to investigate the effect of cocoa extract pre-treatment on product quality and filtration efficiency. The initial aqueous cocoa extract had a pH value of 4.8–5.4 (Peru: 4.8, Cuba: 5.0, Dominican Republic: 5.4). In order to precipitate the protein in the cocoa extract, a bentonite addition was carried out at a temperature of 5 °C and a bentonite quantity of 0 and 200 g/hL at different pH values (3.4, 4.4, 6.4, 7.4, and initial pH value). The experiment was conducted in the same way as described in a previous study [28]. The bentonite (Erbslöh GmbH, Geisenheim, Germany) was swollen in water for 12 h in order to activate it before use.

For each experiment, 16 kg of cocoa extract was homogenized in a container, and the desired pH value was adjusted with an aqueous sodium hydroxide solution (1 N) or anhydrous citric acid (≥99.5%). Then, 200 g/hL bentonite was added. After one hour of incubation at 5 °C, the aqueous cocoa extract was centrifuged using a laboratory centrifuge (3068× *g* for 10 min; type 5810, Vaudaux-Eppendorf AG, Schönbuch, Switzerland). 15 L of the supernatant of the cocoa extract was used for the follow-up filtration experiments.

#### 2.3.3. Filtration of the Supernatant—*Step (II)*

Following the centrifugation, the supernatant was filtered with the CRDM system equipped with ceramic discs, using a variety of ceramic membrane discs with different pore sizes. For each filtration experiment, 15 kg of the pre-treated cocoa extract (supernatant from the pre-treatment, referred to as the feed solution in this step) was filtered. The experiments were performed in a concentration mode to collect the permeate, whereby the feed solution was concentrated to a weight reduction factor (WRF) of 5. After the concentration step, the retentate was then subjected to a further washing step (diafiltration). The aim was to rinse off further compounds from the retentate by adding 4 L of deionized water. To assess the filtration performance, the permeate flux was recorded and evaluated over time using a digital laboratory balance (XS4001S, Mettler Toledo GmbH, Greifensee, Switzerland). The data were recorded on a laptop using the Mettler Toledo BalanceLink (Balance Link Version 4.1.3, Mettler Toledo (Schweiz) GmbH, Greifensee, Switzerland). To assess the quality of the product, the cocoa extract was sampled before and after fining, and the permeate was sampled after the concentration and the diafiltration steps.

Before the membranes were used, they were rinsed with water. The hydraulic permeability ($L_{w}$) of the membranes was determined at four different membrane disc rotation speeds (994, 1128, 1260, 1395 rpm) and three different transmembrane pressures (0.5, 1.0, 1.5 bar) for 50 min in total recycling mode and 10 min in concentration mode, while recording the permeate weight. The flow rate of the feed was set at 100 L/h and the temperature at 30 ± 2 °C. The hydraulic water permeability $L_{w}$ for each test was calculated using Equation (1):

(1) $$L_{w}=\frac{J_{w}}{TMP}$$

with $J_{w}$ representing the water permeate flux at the specific transmembrane pressure (TMP).

With this preliminary test, the best hydraulic permeability results were achieved at a rotation speed of 1260 rpm and a TMP of 0.5 and 1.0 bar (Figure S1, Supplementary Materials).

Although the hydraulic water permeability was highest at 1.0 bar, the permeability of the product was highest at 0.5 bar, so a compromise was made at 0.8 bar, rotation speed of the membrane discs of 1260 rpm and a feed flow rate ($Q_{f}$) of 100 L/h. As it was observed in the preliminary test that the filtrate became cloudy after a short time, the filtration temperature (T) of the tests was set at 10 ± 2 °C. The filtration experiments were carried out using ceramic discs with a pore size of 30 nm, 80 nm, and 200 nm, as these were expected to retain most solids, suspended and colloidal, and proteins without a major loss of polyphenols in the permeate. In a clarification approach for grape marc extract, Mora et al. [34] found that 0.14 µm microfiltration resulted in lower retention of polyphenols compared to pores of 0.8 µm pores, which they explained by the fact that complete pore blockage occurred at 0.8 µm, whereas only intermediate pore blockage occurred in the 0.14 and 0.2 µm membranes. Meanwhile, the retention of suspended solids was higher at 0.14 µm [34].

In addition, Hwang et al. [35] observed that at the same pressure and permeate flux, the blocking index for the 0.4 µm membrane was always higher than that for 0.2 µm-membranes. The blocking index is a key determinant that describes how quickly and to which extent the pores of a membrane are blocked by particles during the filtration process [36]. It is, therefore, important to test different membranes to characterize and optimize which surfaces retain enough proteins and let pass through as many polyphenols as possible.

### 2.4. Dynamic Crossflow Filtration with a Laboratory System (Crossflow Filtration with Rotating Disc Module; CRDM)

The CRDM system contains three flat ceramic discs, each with an area of 0.033 m^2^ and, therefore, an effective filter area of 0.1 m^2^, whereby the ceramic discs are connected to the cylindrical housing. The membrane housing has eight baffles to provide increased turbulence during production operation and, thus, contribute to a better cleaning of the membrane surface. Another advantage of dynamic crossflow filtration is the rotation of the membrane discs, which creates shear and centrifugal forces that prevent the formation of a covering layer and, thus, ensure continuous cleaning of the membranes. The shear rate can be adjusted by changing the rotation speed of the disc. The maximum rotational speed of the membranes is 50 Hz (approx. 1395 rpm); a rotational speed of 1260 rpm (45 Hz) was selected for the experiments. The additional periodic backwashing (pulsing) of the system also maintains a constant filtration performance [37]. The backwash cycle for these experiments was set so that the permeate was backwashed for 5 s every 2.5 min. The product was fed to the filtration system from an external 15 kg tank with a flow rate of 100 L/h using a peristaltic squeeze pump (TP400, Thölen Pumpen GmbH, Geldern, Germany). The transmembrane pressure was set by the pressure valve at the retentate outlet and monitored by the manometer (AISI 316, SUCO Robert Scheuffele GmbH & Co. KG, Bietigheim-Bissingen, Germany) on the membrane housing at a constant level at 0.8 bar.

After filtrating, the system was cleaned with a 2% highly alkaline cleaning agent (calgonit NN 5454, Calvatis AG, Altendorf, Switzerland) and 0.5% cleaning booster with active oxygen (calgonit LAO, Calvatis AG, Altendorf, Switzerland) at a temperature of 50 °C and a pressure of 1.0 bar. The system was circulated for 30 min and then rinsed with tap water until the pH of the permeate was equal to that of tap water.

**Figure 2:**
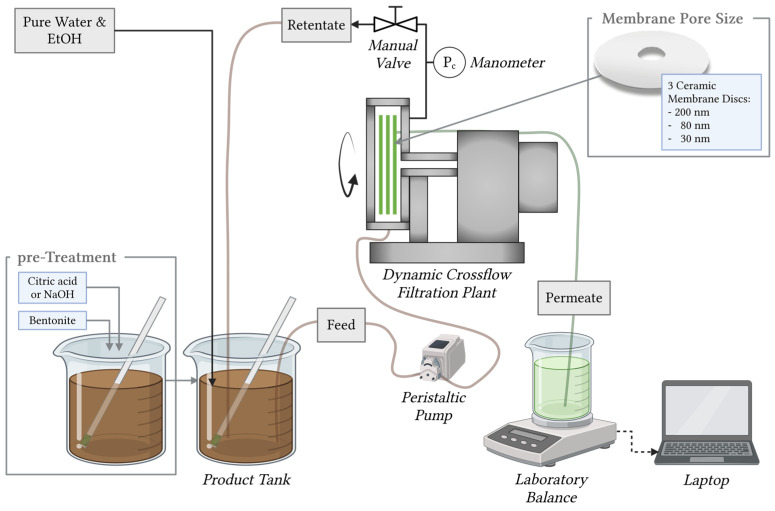
Illustration of the CRDM and the experimental set-up with the dynamic crossflow filtration pilot plant. Feed: Supernatant from pre-treatments; Retentate: retained product; Permeate: clarified product. Created in BioRender [38].

Figure 2 shows the schematic illustration of the CRDM and the experimental setup. Two experimental filtration designs were carried out. Firstly, the influence of pre-treatment was investigated, and, as a second experiment design, the influence of membrane pore size and pre-treatment on filtration productivity and product quality was analyzed.

### 2.5. Evaluation of the Clarification Efficiency of the Filtration Experiments

Filtration performance was evaluated based on the permeation of compounds (permeate quality) and productivity (permeate flux).

#### 2.5.1. Permeate Quality Evaluation

The permeation coefficient (PC) of flavanols, proteins, and dry matter content was estimated by the following equation:

(2) $$PC[\%]=\left(\frac{C_{P}}{C_{F}}\right)\times 100$$

where $C_{P}$ and $C_{F}$ are the absolute concentrations (flavanols, proteins, and dry matter content) in the permeate ($C_{P}$) and initial feed ($C_{F}$) expressed in mg/g.

The performance of the pre-treatment process is expressed as the recovery yield (%) according to the following equation:

(3) $$Y\left[\%\right]=\left(\frac{W_{P}*C_{P}}{W_{F}*C_{F}}\right)\times 100$$

where $W_{F}$ and $W_{P}$ represents the amount of the initial feed and permeate in kg and $C_{F}$ and $C_{P}$ represents the concentration of the respective compounds in the initial feed and permeate.

#### 2.5.2. Productivity Evaluation

The permeate flux, $J_{P}$, was determined by recording the collected permeate weight during a certain time through the membrane surface using the following equation:

(4) $$J_{P}=\frac{W_{P}}{\left(A\times t\right)}$$

where $J_{P}$ is the permeate flux (kg/m^2^h), $W_{P}$ is the weight of the collected permeate (kg) at the time t (h) and A is the effective membrane area (m^2^).

Weight reduction factor (WRF) is defined as the ratio between the initial feed weight and the weight of the resulting retentate according to the following equation:

(5) $$WRF=\frac{W_{F}}{W_{R}}$$

where $W_{F}$ and $W_{R}$ are the initial feed weight and retentate weight in kg, respectively.

## 3. Results and Discussion

### 3.1. Influence of the Pre-Treatment of Cocoa Extract on the Yield in Relation to the Analysed Compounds

**Figure 3:**
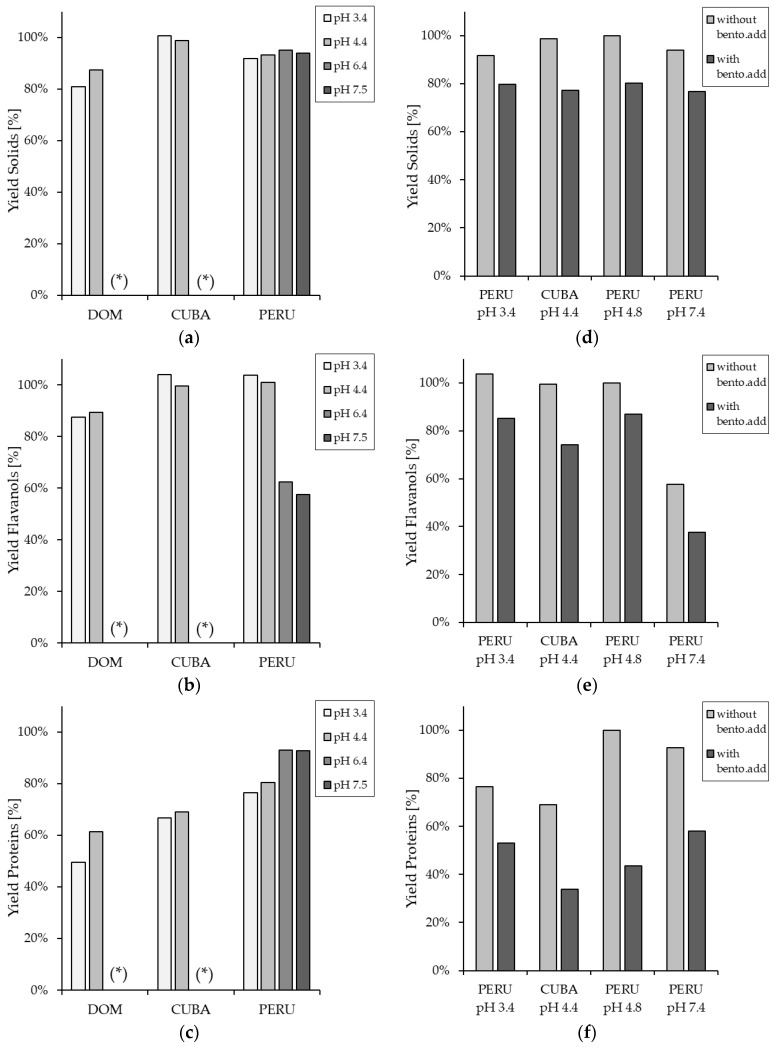
Yield after pre-treatment of cocoa extracts in terms of solids expressed as dry matter content (**a**), flavanols (**b**), and protein contents (**c**) in the supernatant. And the yield after pre-treatment experiments with and without bentonite addition (bento.add) in terms of solids (**d**), flavanols (**e**), and proteins (**f**) in the supernatant. If marked with (*), no data is available.

In order to determine the influence of pH modification and the addition of bentonite on the analyzed constituents of the cocoa extract after pre-treatment, the experiments described in Section 2.3.2 were carried out with different pH values (3.4, 4.4, 6.4, 7.4, and the initial pH value) and the addition of the fining agent bentonite (0 and 200 g/hL). As shown in Figure 3, the changes from the initial pH value to pH 6.4 and 7.4 resulted in only a slight removal of the proteins of about 5–10%. On the other hand, a change of pH to 4.4 resulted in a more intense decrease in protein yield in the pre-treated cocoa extract from all origins, reaching its maximum at pH 3.4 when the pH was lowered further due to the formation of aggregates, which precipitate during centrifugation. Cerit et al. [39] obtained similar results in their study. They changed the pH in their cocoa extracts to precipitate the proteins and found that protein precipitation was highest at a pH of 3.5 due to the pH change with phosphoric acid [39]. Loginov et al. [40] also investigated the effect of changing pH during flaxseed hull extraction and found that the precipitation was obviously most efficient at the isoelectric point of cocoa proteins due to the lack of electric repulsion between uncharged protein molecules [39,40].

Furthermore, Loginov et al. [40] observed that the proteins were not completely removed by centrifugation, even at the isoelectric point, due to the presence of smaller proteins, proteins with varying isoelectric points, or denatured proteins of different solubilities in the extracts. This agrees with the results of the present study, where it was also observed that the whole protein content could not be precipitated at the isoelectric point and fully removed by centrifugation. Obviously, smaller residual proteins cannot be centrifuged. In addition, the amount of protein removal by the pre-treatment process varies depending on the origin of the cocoa extract.

When the pH value was changed to 3.4 and 4.4, the flavanol yield after centrifugation changed only slightly by about 0–15%. Again, depending on the origin of the cocoa extract, the decrease in flavanols due to the pre-treatment process varies. However, when the pH value was changed from the initial pH to pH 6.4 and 7.4, a noticeable decrease in the flavanols of about 40% was observed in the cocoa extract. An increase in the removal of polyphenols by centrifugation with increasing pH was also observed by Loginov et al. [40]. Their explanation, while being typical textbook protein chemistry knowledge, was that lowering the pH suppresses their dissociation and forces protonation of especially phenolic acids, increasing the hydrophobic interaction of polyphenols with the proteins present in the extracts [40]. Dangles [41] described polyphenols as weakly acidic compounds, whereby they are neutral in an acidic medium and form anions in a strongly basic medium. Loginov et al. [40], therefore, suggested that adsorption on the surface of protein aggregates could explain the partial removal of polyphenols in coagulated extracts during centrifugation (in their studies at a pH ≤ 9.0). In another study described by Scalbert et al. [42], it was found that mild alkaline hydrolysis releases ester-bound polyphenols, whereas acid hydrolysis releases ether-bound polyphenols from wheat straw. The same observation has also been made in several other studies, where it was reported that both acidic and basic extraction lead to a release of bound basic and acidic phenolic compounds, respectively [43,44,45,46]. Furthermore, in their study on the stability of tea polyphenols, Zeng et al. observed that tea polyphenols with a pH value of 3–6 remained stable at 4 and 25 °C, while the higher the pH, while the higher the pH value, the more unstable the tea polyphenols become [47].

When, in addition to the change in pH, the fining agent bentonite is added, a decrease in both components can be observed at all pH values and from all origins of the cocoa extracts. It can, therefore, be assumed that the addition of bentonite precipitates not only proteins but also a certain amount of flavanols, especially the ones with a higher DP. Similar results were observed in a recent study by Beeler et al. [28] and in a study described by He et al. [48], where the addition of bentonite to white wine resulted in a significant decrease in protein content but also a decrease of total phenolic content. It can also be observed that the protein yield decreases when the pH is lowered to 3.4. This can be explained by the fact that bentonite fixes the proteins at an optimum ambient pH (pH 3.0–3.3) [26].

**Figure 4:**
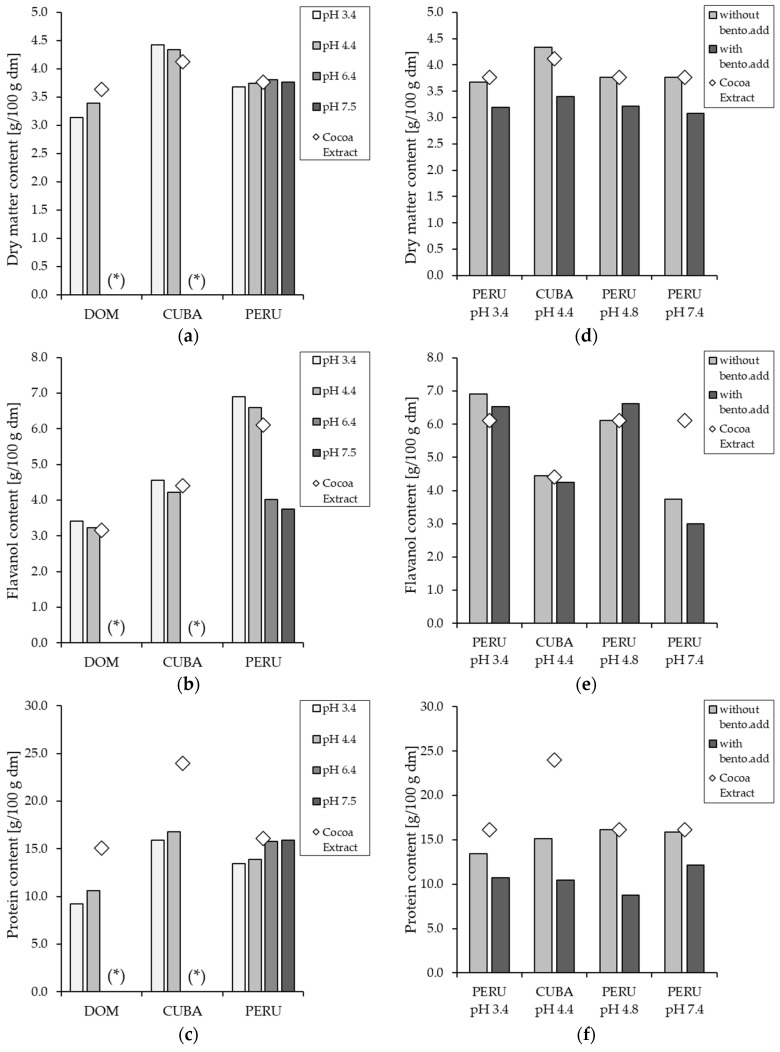
Dry matter (**a**), flavanol (**b**), and protein content (**c**) in g/100 g dry matter (dm) of the cocoa extract and supernatant after pre-treatment. And the content of the cocoa extract and supernatant after pre-treatment experiments with and without bentonite addition (bento.add) in terms of solids (**d**), flavanols (**e**), and proteins (**f**). If marked with (*), no data is available.

A similar phenomenon is obvious from Figure 4, which shows the flavanol content in the supernatant after pre-treatment and centrifugation, as it is important to understand if there is a higher flavanol content in order of the diminished protein content. As for the yield, the flavanol content was highest at pH 3.4, followed by pH 4.4, the initial pH (4.8–5.4), pH 6.4, and the lowest at pH 7.4. Although the pre-treatment with pH modification and bentonite addition led to a decrease in the flavanol yield after centrifugation (Figure 3), an enrichment of the flavanols was achieved in the supernatant, except for the samples with pH 6.4 and 7.4. Furthermore, the flavanol content of the cocoa samples from Peru was the highest, followed by Cuba and the Dominican Republic, with the lowest content. In addition, the protein content in all extracts was diminished by the pre-treatment. The decrease in protein content was the opposite of that of flavanols, with the lowest content at pH 3.4 and an increase up to pH 7.4. The dry matter content shows similar behavior to the protein content, decreasing from pH 7.4 to 3.4, except for the Cuban samples, where the dry matter content was higher at pH 3.4 than at pH 4.4.

### 3.2. Influence of Different Membrane Pore Sizes of the Filtration on the Permeate Quality and Productivity

**Figure 5:**
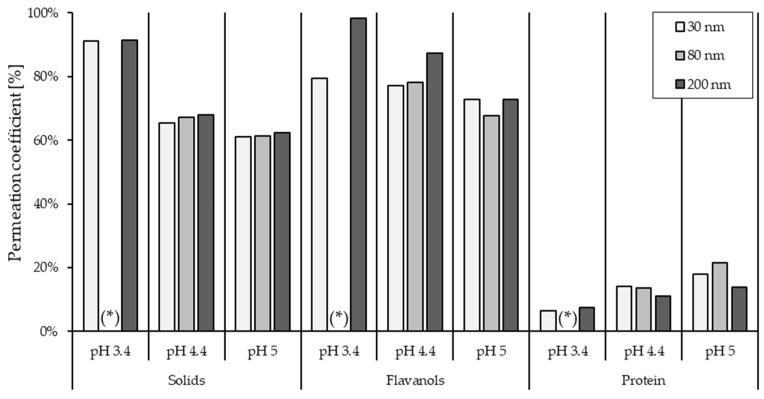
Permeation coefficient of cocoa solids expressed as dry matter content, flavanol, and protein content of the cocoa extract after pre-treatment and filtration with the origin from Cuba. If marked with (*), no data is available.

The cocoa extracts’ supernatants obtained from the pre-treatment were also used as feed material in the experiments to determine which membranes should be used for the purification process in terms of membrane performance. Figure 5 shows the influence of the three different membrane pore sizes (30 nm, 80 nm, and 200 nm) in terms of the permeation coefficient of flavanols and proteins. The highest permeation of flavanols was obtained by filtration with a pore size of 200 nm. This was achieved at a pH value of 5.0 as well as at 3.4 and 4.4. In contrast to the highest permeation of flavanols in the permeate, a lower yield of proteins was observed with the 200 nm pore size compared to the other pore sizes. The lower yield at 200 nm at pH 5.0 and pH 4.4 or higher at pH 3.4 does not appear to show a clear pattern or significance. Therefore, it cannot be concluded that there is a major difference between these membrane pore sizes in protein permeation. Cocoa solids, on the other hand, showed similar behavior to flavanol permeation for all three membrane pore sizes.

Wang et al. [49], who purified polyphenols from apple skin, found similar results. In their study, they also observed a significant reduction in the permeation of polyphenols as well as proteins, the smaller the membrane pore size. This phenomenon can be primarily explained by the influence of the large molecular size of the protein molecules [49]. Chandini et al. [50] also observed that the permeation of polyphenols was higher with a larger membrane pore size on the purification of tea extracts [50]. However, this is not consistent with the results of Mora et al. [34], who found that a higher rejection was achieved with 0.8 µm than with 0.2 and 0.14 µm, which they explained with different fouling mechanisms.

**Figure 6:**
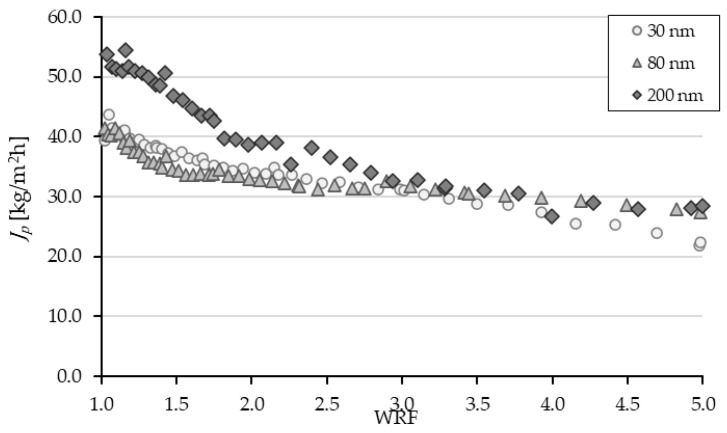
Permeate flux ($J_{P}$) in kg/(m^2^h) as a function of the WRF at three different membrane pore sizes. Operation conditions: TMP 0.8 bar; Q_f_, 100 L/min; T, 10 °C.

In addition to permeate quality, the permeate flux with a 200 nm membrane pore size at pH 5.0 showed the best results (Figure 6). It can be seen that the permeate flux was best with the 200 nm pore size. Based on these results, further filtration experiments were carried out to investigate the influence of pre-treatment of the cocoa extract, followed by filtration using a ceramic membrane with a pore size of 200 nm on the flavanol content.

### 3.3. Influence of Pre-Treatment and Filtration on the Permeate Quality and Productivity

**Figure 7:**
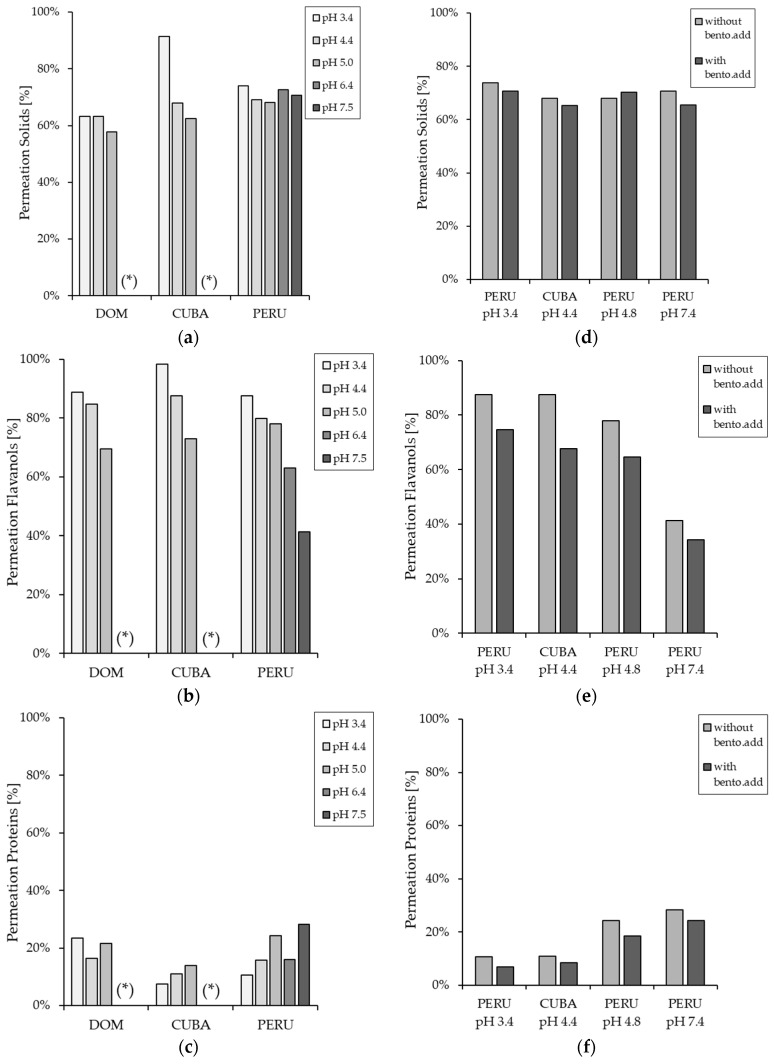
Permeation coefficient of cocoa solids expressed as dry matter content (**a**), flavanols (**b**), and proteins (**c**) after pH-modification and filtration. As well as permeation of the cocoa solids (**d**), flavanols (**e**), and proteins (**f**) after pH modification with and without bentonite addition (bento.add) and filtration. If marked with (*), no data is available.

In order to investigate the influence of both pre-treatment and filtration on the yield of flavanols and proteins in terms of permeation coefficient, the supernatants of the pre-treated cocoa extracts after centrifugation were also used for filtration experiments with the CRMD. Figure 7 shows that changing the initial pH value to 4.4 and 3.4, followed by 200 nm filtration, resulting in a higher permeation of flavanols in the permeate compared to the initial pH value for all origins. On the other hand, the permeation of flavanols in the permeate was reduced by increasing the pH to 6.4 and 7.4.

Also, Conidi et al. [46] observed a similar phenomenon, where an increase in the pH of bergamot juice led to increased retention of polyphenols during ultrafiltration.

They explained this by the fact that when the pH value is increased, the polyphenols precipitate and form a filter cake on the membrane surface [46]. Chethan et al. [47] found that polyphenols are very sensitive to changes in pH; even a small change in pH by adding sodium hydroxide solution led to the formation of precipitates, which increased with a further increase in pH of 10. In addition, it was found that the precipitation was reversible, as it was completely dissolved when acid was added. However, this is obvious from traditional polyphenol chemistry. Additionally, under such conditions, polyphenols can polymerize and form melanins, or they can crosslink proteins, forming reaction products of varying solubility, altered brown color, or further properties [7].

In a model study with BSA, Howell et al. [51] observed that maximum protein retention occurs at a pH corresponding to the isoelectric point of the proteins. The lower permeability of the proteins is explained by an increased attraction of uncharged proteins to the filtration membrane and the formation of a thicker or denser fouling layer [51,52]. Even after filtration, the addition of bentonite resulted in a similar phenomenon to that observed in the supernatant after pre-treatment. Again, the addition of bentonite not only reduced the amount of proteins in the permeate but also reduced the amount of flavanols in the permeate.

**Figure 8:**
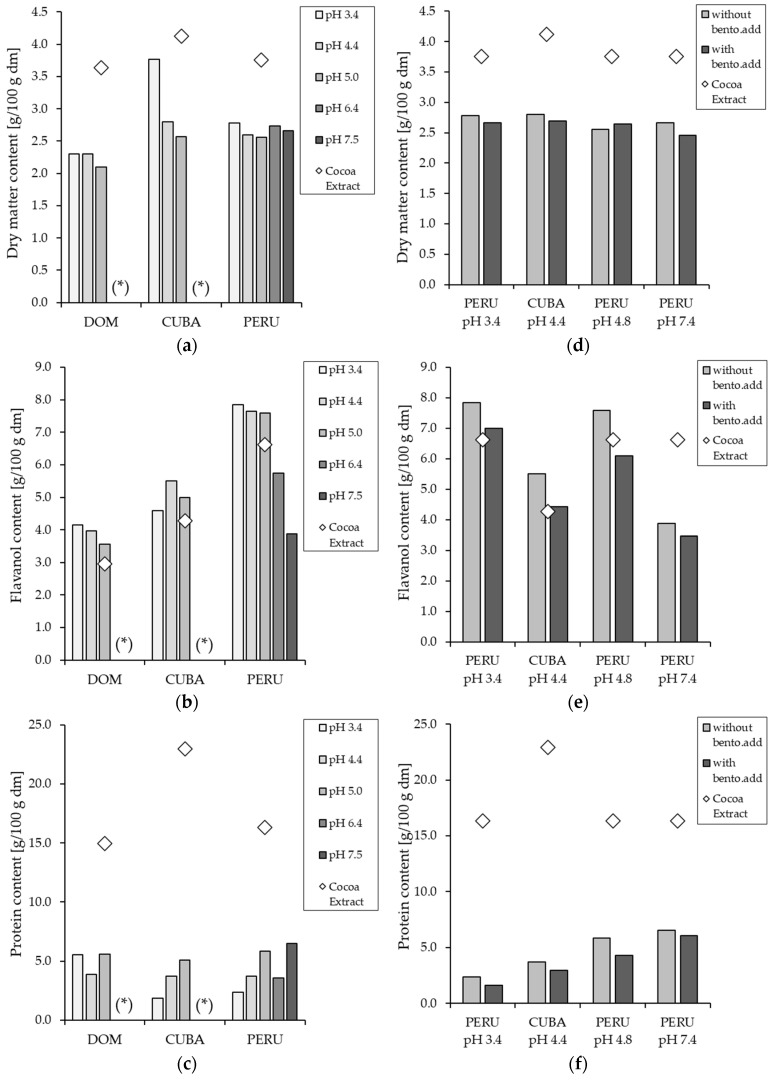
Dry matter (**a**), flavanol (**b**), and protein content (**c**) in g/100 g dry matter (dm) of the cocoa extract and permeate after pre-treatment and filtration. And the content of the cocoa solids (**d**), flavanols (**e**), and proteins (**f**) of the cocoa extract and permeate after pH-modification with and without bentonite addition (bento.add) and filtration. If marked with (*), no data is available.

The quality of the permeate, as well as the purification effect of the pre-treatment and the filtration process, were evaluated. Figure 8 shows the flavanol content in the permeate compared to the initial flavanol content in the cocoa extract. For all three origins, the flavanol content in the permeate was increased by the two-step process, except for the experiments with pH 6.4, pH 7.4, and the experiments with bentonite addition. Furthermore, the decrease of the initial pH value to 4.4 and 3.4 not only increased the permeation in the permeate but also resulted in a higher enrichment. In terms of protein content, it can be seen that the additional filtration after the pre-treatment has resulted in a further diminishing of the protein content. As with the pre-treatment results, lowering the pH from 7.4 to 3.4 leads to a decrease in protein content, with the permeate at pH 3.4 having the lowest protein content except for the permeate from the Dominican Republic. This could be due to the fact that the isoelectric point at 3.5 of the proteins in the cocoa extract. A similar phenomenon was observed by Zhu et al. [53] in their study, which they attributed to a change in pH, which can alter the net charge of the protein and tends to alter the electrostatic interactions between protein-membrane and protein-protein. Therefore, pH changes towards the isoelectric point result in a higher tendency for protein adsorption and aggregation on the membrane surface and, thus, higher filtration quality [53].

**Figure 9:**
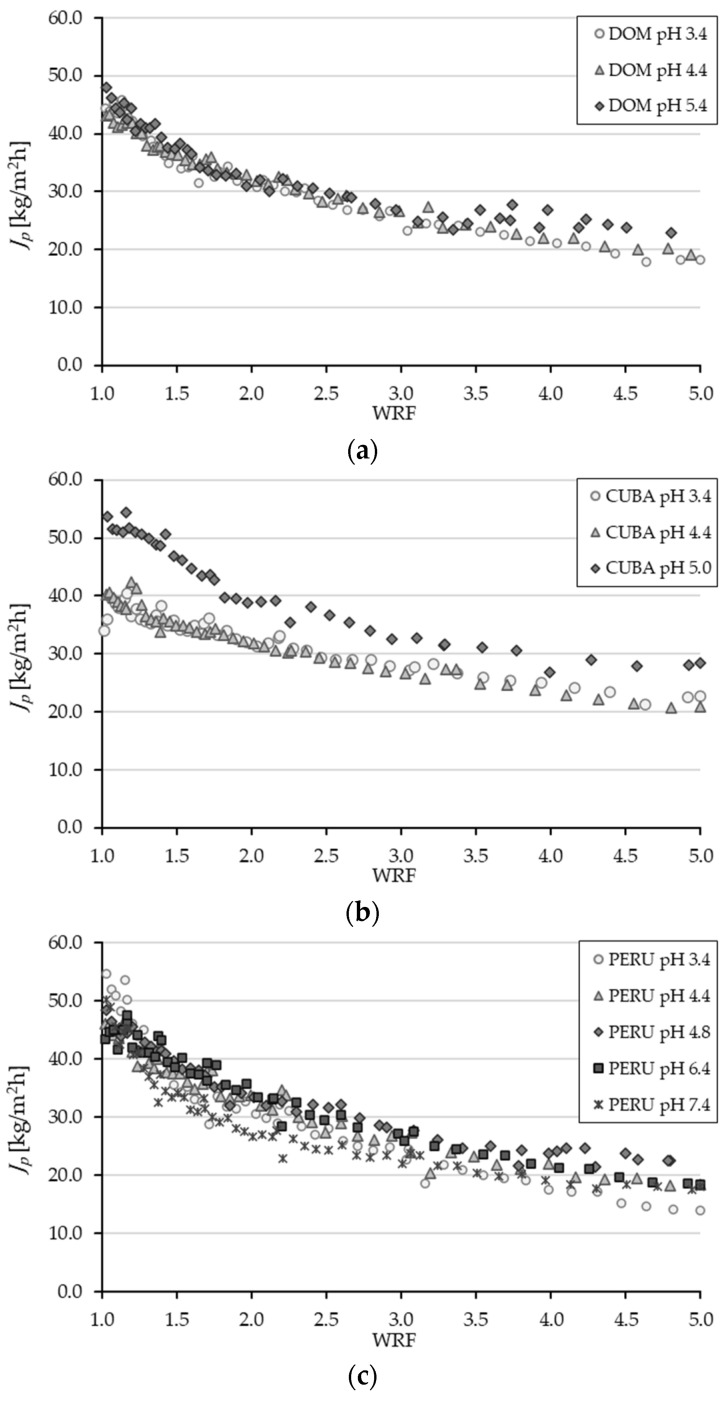
Permeate flux ($J_{P}$) in kg/(m^2^h) as a function of the WRF at five different pH values for (**a**) the origin DOM, (**b**) the origin CUBA, and (**c**) the origin PERU. Operation conditions: TMP 0.8 bar; Q_f_, 100 L/min; T, 10 °C.

In addition to the permeate quality, filtration productivity in terms of the permeate flux also plays an important role. Figure 9 shows the permeate flow as a function of WRF. It is noticeable that the permeate flow decreases accordingly with decreasing pH, but no statistically significant difference could be determined. Papaioannou et al. [54] also observed that acidic pH filtration had a negative effect on the efficiency of the UF process by reducing permeability during the valorization of pomegranate husk. This is because the change in pH causes a change in the charge of the dissolved organic compounds [54]. Tanudjaja et al. [55] describe three stages of membrane fouling and the decrease in permeate flux. Initially, a reversible concentration-polarization boundary layer is quickly formed on the surface of the membrane, which reduces the driving force for filtration. Secondly, proteins adsorb or accumulate in the pores of the membrane. Finally, there is a quasi-stationary flow of the permeate [55]. It is, therefore, assumed that lowering the pH towards the isoelectric point of the proteins causes more proteins to adsorb onto the membrane surface, thereby decreasing the permeate flux. Sarkar et al. [56] observed that lowering the pH to a value corresponding to the isoelectric point of the proteins led to strong fouling during filtration in the recovery of proteins from whey for the separation of the whey proteins α-lactalbumin and β-lactoglobulin. As a result, permeate flux is lowest at the specific pH value [56].

Loginov et al. [40] also investigated the influence of pH adjustment on the filterability and separation, or selectivity, of polyphenols and proteins using flaxseed hull extract. They found that a decrease in pH from 13.2 to 4.4 resulted in a large decrease in permeate flux but also lower retention and better purity of the polyphenols. This is due to the fact that membrane fouling—caused by the increased attraction of uncharged proteins due to the low pH—resulted in improved separation of polyphenols and proteins and, thus, improved permeate quality [40]. However, a fouling of the membrane leads to a higher quality of the permeate due to the increased selectivity, as was also observed by Zhu et al. [53] and Loginov et al. [40].

## 4. Conclusions

When trying to isolate polyphenols from aqueous cocoa extracts, it is important to consider that various factors, such as yield and the stability of the polyphenols, play a crucial role. Additionally, the presence of residual proteins must be considered, as they can interfere by binding to polyphenols or forming complexes/adducts, which can reduce the yield, purity, and properties of the isolated compounds. To enrich flavanols from cocoa extracts, it may be necessary to remove proteins and other compounds. However, binding (to proteins or other biomacromolecules) and the interactions behind it are quite complex and can occur in the different steps of the whole process, from extraction over precipitation to filtration, which is why there is a need for optimization.

In the present study, pre-treatment with a pH modification showed that reducing pH from 5.0 to 4.4, and maximally at 3.4, decreased protein content, while higher pH levels (6.4 and 7.4) yielded only a slight protein reduction (5–10%). Flavanol content changed minimally (0–15%) when pH was adjusted from 5.0 to 3.4 and 4.4, in contrast, it decreased by about 40% when pH was increased to 6.4 and 7.4.

The addition of the additive bentonite during pre-treatment decreased both proteins and flavanols at all pH values and cocoa extract origins, indicating that bentonite precipitates both proteins and some flavanols, particularly those with high degrees of polymerization. Reducing the initial pH to 4.4 and 3.4 during filtration not only increased flavanol permeation but also resulted in higher enrichment, consistent with pre-treatment results. The addition of bentonite during filtration similarly reduced both protein and flavanol content in the permeate.

This study indicates the promising potential of a combined pre-treatment and filtration process for polyphenol enrichment. Furthermore, the results of this study promise good yields at a pH range close to the isoelectric points of the cocoa proteins and a recommended 0.2 µm filtration size. However, further investigations are needed to investigate the accumulation and potential aggregation of the flavanols of the aqueous cocoa extract. These findings may be applicable to other complex polyphenol-rich extracts and a promising approach to developing polyphenol extraction processes when sustainability is also of interest, and the value of isolated polyphenols can contribute to an attractive exploitation.

## Data Availability

The data presented in this study are available on request from the corresponding author.

## Supplementary Materials

The following supporting information can be downloaded at https://www.mdpi.com/article/10.3390/membranes14110242/s1, Figure S1: (a) hydraulic water permeability L_w and (b) product permeability with a membrane pore size of 0.2 µm expressed in kg/(m^2^ × h × bar) as a function of the rotation speed of the membrane disc in rpm. Table S1: Information on the calibration and detection parameters that are used for the analysis of the different compounds that are quantified, Figure S2: Chromatogram of the NIST cocoa flavanol standard to illustrate the DP groups.
