# Supplementary material for: Concentrating Cocoa Polyphenols—Clarification of an Aqueous Cocoa Extract by Protein Precipitation and Filtration
Source: Membranes (Basel). 2024 Nov 17;14(11):242. doi: 10.3390/membranes14110242 (PMC11596179; doi:10.3390/membranes14110242)
Supplement: Supplementary file 1 [file membranes-14-00242-s001.zip › membranes-3267828-supplementary.pdf]

Supplementary Materials

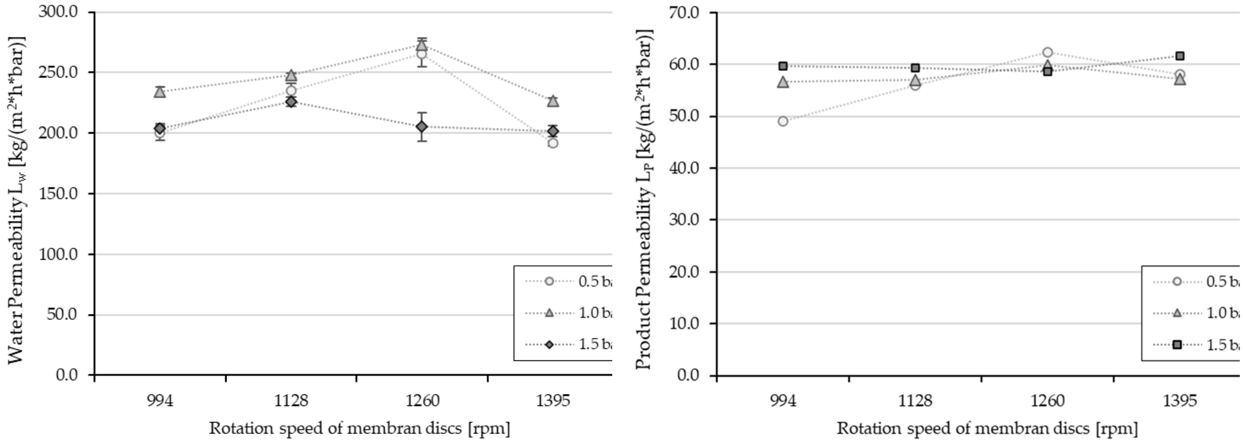

(a) **(b)**

Figure S1. (**a**) hydraulic water permeability L_w and (**b**) product permeability with a membrane pore size of 0.2 µm expressed in kg/(m^2 × h × bar) as a function of the rotation speed of the membrane disc in rpm.

Table S1. Information on the calibration and detection parameters that are used for the analysis of the different compounds that are quantified.

Group	Retention Time Start	Retention Time Stop	Measurement-/ Detection System	Detection Parameters	Calibration Points	Calibration Range [µg/mL]	Calibration Line	(r^2)
DP1	0.900	1.790	UPLC-FLD	Ex = 230 nm Em = 321 nm	5	5.102 – 25.508	y = 1.02e+05x-1.27e+05	0.999174
DP2	1.800	3.700	UPLC-FLD	Ex = 230 nm Em = 321 nm	5	3.667 – 18.337	y = 6.12e+04x-1.08e+05	0.999286
DP3	3.710	5.300	UPLC-FLD	Ex = 230 nm Em = 321 nm	5	3.881 – 19.403	y = 2.04e+04x-3.48e+04	0.999883
DP4	5.310	6.400	UPLC-FLD	Ex = 230 nm Em = 321 nm	5	3.286 – 16.438	y = 2.57e+04x-3.07e+04	0.999480
DP5	6.410	7.300	UPLC-FLD	Ex = 230 nm Em = 321 nm	5	2.784 – 13.919	y = 2.16e+04x-2.32e+04	0.996784
DP6	7.310	7.950	UPLC-FLD	Ex = 230 nm Em = 321 nm	5	2.149 – 10.745	y = 1.63e+04x-1.11e+04	0.996907
DP7	7.951	8.500	UPLC-FLD	Ex = 230 nm Em = 321 nm	5	1.629 – 8.147	y = 9.92e+03x_3.32e+03	0.987352

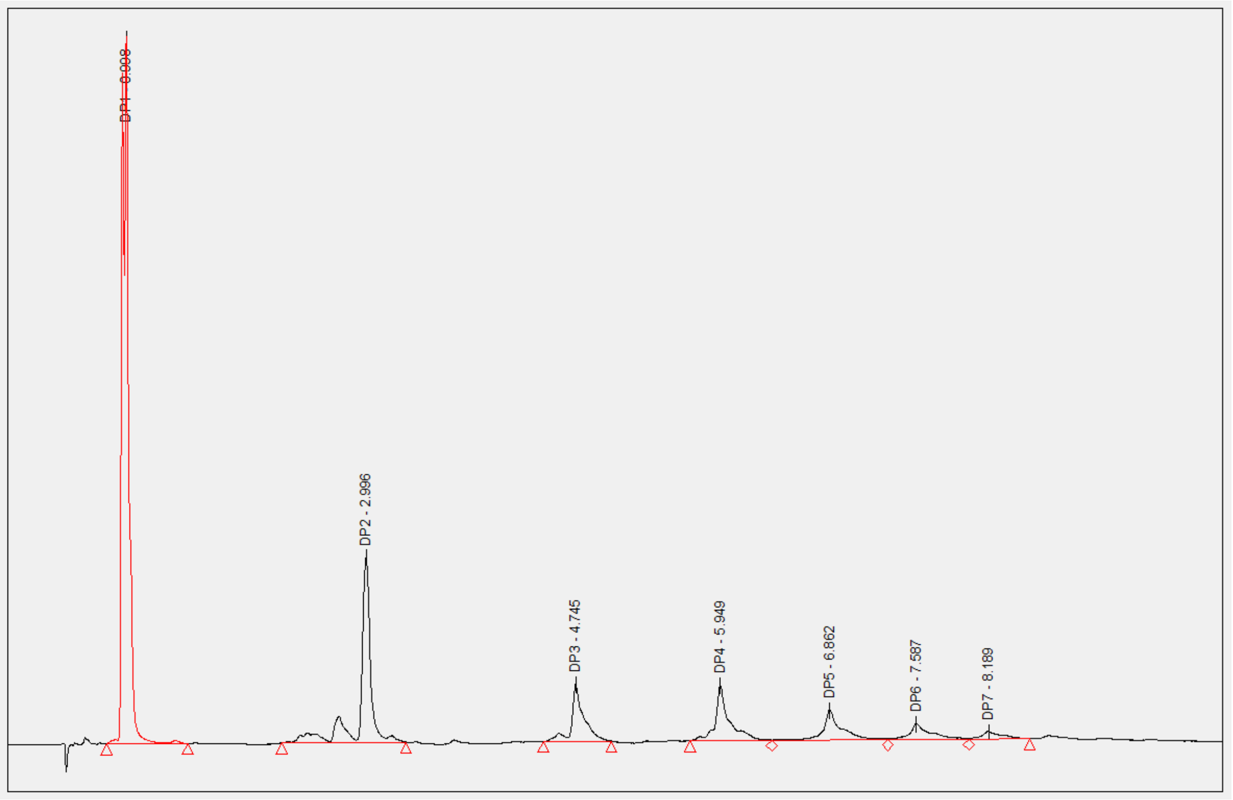

Figure S2. Chromatogram of the NIST cocoa flavanol standard to illustrate the DP groups.
